# A Study to Validate the Relevance of Semen F_2_-Isoprostanes on Human Male Infertility

**DOI:** 10.3390/ijerph19031642

**Published:** 2022-01-31

**Authors:** Elena Moretti, Cinzia Signorini, Fabio Ferretti, Daria Noto, Giulia Collodel

**Affiliations:** 1 Department of Molecular and Developmental Medicine, University of Siena, Policlinico Le Scotte, Viale Bracci, 14, 53100 Siena, Italy; elena.moretti@unisi.it (E.M.); noto@student.unisi.it (D.N.); giulia.collodel@unisi.it (G.C.); 2 Department of Medical and Surgical Sciences and Neurosciences, University of Siena, Policlinico Le Scotte, Viale Bracci, 14, 53100 Siena, Italy; ferrefa@unisi.it

**Keywords:** idiopathic infertility, male infertility, seminal F_2_-isoprostanes, sperm motility, varicocele, urogenital infections

## Abstract

F_2_-isoprostanes (F_2_-IsoPs), byproducts of arachidonic acid oxidation, are one of the most reliable indices for assessing lipid peroxidation in vivo. This study aimed at evaluating the seminal F_2_-IsoP level in 147 patients with different reproductive conditions (varicocele, urogenital infection, idiopathic infertility) and 45 fertile controls to establish a cut-off value discriminating physiological and pathological ranges. Semen analyses were performed following WHO guidelines; F_2_-IsoP levels were measured by gas chromatography/negative-ion chemical ionization tandem mass spectrometry. Considering the whole group of patients, F_2_-IsoPs correlated negatively with normal morphology (r = −0.283, *p* < 0.01), viability (r = −0.245, *p* < 0.01), total progressive motility (r = −0.309, *p* < 0.01) and rapid motility (r = −0.535, *p* < 0.01). The area under the ROC curve for F_2_-IsoP levels was 0.839, indicating a good performance of the test; the Youden index showed a cut-off value of 29.96 ng/mL. Fertile men (except one) were distributed in the group of patients with F_2_-IsoP level < 29.96 ng/mL. Varicocele and urogenital infection groups showed the highest levels of F_2_-IsoPs in semen. For the first time, a cut-off for F_2_-IsoPs is identified in human semen. It allows discriminating different male infertility conditions by the semen F_2_-IsoP amounts, as an additional parameter for clinical evaluation.

## 1. Introduction

As a series of prostaglandin (PG)-like compounds, F_2_-isoprostanes (F_2_-IsoPs) are produced by the free radical-catalyzed peroxidation of the polyunsaturated arachidonic acid [1]. Lipid peroxidation (LPO) is a critical effect of oxidative stress, which is a common biological condition in a wide range of human diseases such as neurological disorders, cardiovascular diseases, diabetes and renal dysfunction [2]. F_2_-IsoPs have been indicated as a reliable biomarker of LPO in human pathological conditions [3,4,5,6]. F_2_-IsoPs are first formed as molecules esterified in phospholipids and subsequently released in an unesterified form (free F_2_-IsoPs). For the purpose of F_2_-IsoP quantification, the most widely measured isomer is 8-iso-PGF_2α_ [7]. Nevertheless, evidence suggested that the free form of 8-iso-PGF_2α_ has significant limitations as a measure of oxidative damage since it is also produced by enzymatic activity [6]. Remarkably, the evaluation of total 8-iso-PGF_2α_ amounts, used in this research, is less affected by enzymatic metabolism because esterified arachidonic acid is not a substrate for prostaglandin-endoperoxide synthases [6].

The quantification of F_2_-IsoPs in biological fluids may be carried out by different analytical methods such as gas chromatography–mass spectrometry, liquid chromatography–mass spectrometry and immunoassays (ELISAs) [7]. In particular, F_2_-IsoPs have been measured in mammalian blood plasma, urine, cerebrospinal fluid, sputum, saliva, exhaled breath condensate, brain tissues, atherosclerotic plaques, gastric mucosa and, recently, also in human semen [7].

The relevance of F_2_-IsoPs in human diseases is related not only to the evaluation of oxidative stress occurrence, but also to their potential biological activity, as they are considered mediators of oxidative damage [7]. Taber et al. [8] reported that 8-iso-PGF2α has a vasoconstrictive action on smooth muscle cells by using receptors other than those of thromboxane [9,10]. Moreover, the expression of endothelin-1, which causes endothelial cell alterations, is stimulated by F_2_-IsoPs released during oxidative damage [11].

It is known that spermatozoa are rich in polyunsaturated fatty acids (PUFAs) and susceptible to LPO, which limits their fertilizing potential. Recently, we detected increased F_2_-IsoP levels in semen of patients affected by varicocele, leukocytospermia, idiopathic infertility and genetic sperm defects compared to fertile men, suggesting that they represent a shared feature in different pathological conditions [12,13,14].

It has been also observed that a mild increase in F_2_-IsoP levels was related to DNA integrity and good embryo quality, suggesting that different amounts of seminal F_2_-IsoPs could have variable effects on sperm metabolic activity [15].

This study aimed at evaluating, in a large group of patients with different reproductive conditions, the levels of total (free plus esterified) seminal F_2_-IsoPs to identify a cut-off point discriminating physiological and pathological values of these compounds. In addition, the relationship between the detected amount of isoprostanoids and sperm parameters was evaluated. 

## 2. Materials and Methods

### 2.1. Patients

We enrolled 147 patients (aged 26–43 years) with infertility attending our laboratory for semen analysis in the period between January 2018 and December 2019. All patients were unable to obtain pregnancy after 2 years of unprotected sexual intercourse. The female factor was excluded.

The infertile patients were categorized according to clinical diagnosis: group with genitourinary infection (n = 52), group with varicocele (n = 54), group with idiopathic infertility (n = 41).

The inclusion criteria common for the three groups were as follows: nonazoospermic and nonobese patients with a normal 46, XY karyotype; a normal hormonal profile (FSH, LH and T); no history of radiotherapy, chemotherapy, chronic illness or medication; the absence of systematic sperm defects; and the absence of cryptorchidism. In addition, they were nonsmokers and had no alcohol dependence; none of them was taking an oral antioxidant supplement for 5 months before the analysis.

The patients included in the varicocele group showed both physical examination and scrotal color Doppler ultrasonography analysis performed in different laboratories other than ours. The occurrence of clinically asymptomatic genitourinary infection was assayed by a bacteriological analysis; patients were considered infected if they showed positive bacteriological cultures. The patients with both varicocele and positive semen bacteriology were excluded from the analysis, as were patients with leukocytospermia.

In the group of genitourinary infections, 20 semen samples were positive for *Enterococcus faecalis*, 14 for *Escherichia coli*, 8 for *Streptococcus agalactiae*, 4 for *Ureaplasma urealyticum*, 2 for *Staphylococcus aureus*, *2* for *Micoplasma hominis*, 1 for both *Escherichia coli* and *Streptococcus agalactiae* and 1 for both *Escherichia coli* and *Enterococcus faecalis*.

In the varicocele group, left-sided varicocele was found in 38 cases (11 patients with grade III, 19 patients with grade II and 8 with grade I) and right-sided varicocele and bilateral varicocele were diagnosed in 10 (grade II) and 6 (grade I–II) patients, respectively.

In the group with idiopathic infertility, we included patients who had no history of diseases affecting fertility and, despite having normal physical, endocrine, genetic and biochemical laboratory values, showed abnormal sperm parameters.

As controls, a fertile group of 45 men with proven fertility having fathered at least one child in the past 3 years was considered. Fertile men (aged 24–41) with normal karyotype were not affected by anatomical problems and/or infections. The patients provided informed consent for this research as required by the Ethics Committee of Azienda Ospedaliera Universitaria Senese, CEAOUS. The patients were guaranteed that the semen was used for the approved protocol and not used for any assisted reproduction techniques. 

### 2.2. Light Microscopy

Semen samples were collected by masturbation in a sterile container; a part of each semen sample, recovered with sterile pipettes, was sent to the microbiological laboratory (within 1.30 h after collection) as advised in WHO guidelines [16]. The peroxidase stain was used for leukocyte identification; a value of >1 × 10^6^ leukocytes/mL was considered out of range.

For semen analysis, samples were examined after liquefaction for 30 min at 37 °C. Volume, pH, sperm concentration, progressive motility (rapid and slow), normal morphology and viability were assessed as recommended by WHO [16]. 

### 2.3. F_2_-IsoP Determination

F_2_-IsoPs are initially produced in situ on phospholipids and defined as esterified F_2_-IsoPs; finally, such esterified compounds are released into the circulation as free (not esterified) F_2_-IsoPs. Here, total (free plus esterified) F_2_-IsoPs were quantified in semen plasma by gas chromatography/negative-ion chemical ionization tandem mass spectrometry (GC/NICI-MS/MS) analysis. In particular, butylated hydroxytoluene (BHT) was added (final concentration 90 μM) to each sperm sample at the time of collection. At this stage, storage at −80 °C could be carried out until the assay time. At the time of F_2_-IsoP detection, in each sample, a basic hydrolysis was firstly performed by incubation (45 °C, 45 min) with 1 N KOH (1:0.5, *v*:*v*). Afterward, sample acidification was obtained by adding HCl 1 N (1:0.5, *v*:*v*), and a tetradeuterated derivative of prostaglandin (PGF_2α_-d4, 500 pg) was added as an internal standard. Sample purification was carried out by two different solid-phase extractions (octadecylsilane, C18 cartridge, and aminopropyl, NH_2_ cartridge) to obtain a final eluate to be derivatized before the GC/NICI-MS/MS was performed [17]. The amounts of 8-iso-PGF_2α_, the most represented isomer for F_2_-IsoP detection (also known as 15-F_2t_-IsoP), were quantified by measuring *m*/*z* 299 product ion, derived from the [M-181]^−^ precursor ions, and compared to the *m*/*z* 303 ion produced by PGF_2α_-d4 in the applied GC/NICI-MS/MS [17]. For quantification, a calibration curve was constructed using reference 8-iso-PGF_2α_ compound (Cayman Chemical, Item No. 16350).

### 2.4. Statistical Analysis

All analyses were performed by IBM SPSS Statistics Software v.25.

The Kolmogorov–Smirnov test was used to verify the normality in the distribution of the variables. The variables resulted not normally distributed and data were reported as median and interquartile range (IQR).

Spearman coefficient was calculated to measure the correlation between variables for all enrolled patients. The comparisons between groups (fertile, idiopathic infertility, urogenital infections, varicocele) were evaluated by Kruskal–Wallis test, followed, only for significant cases, by Dunn’s post hoc test for multiple comparisons.

The receiver operating characteristic (ROC) curve procedure was calculated to describe the accuracy of F_2_-IsoP measurement in terms of the relationship between sensitivity and specificity. The Youden index J, a measure of overall diagnostic effectiveness and a function of sensitivity and specificity, was used to establish the cut-off value for F_2_-IsoP level. J falls at the cutpoint that optimizes the F_2_-IsoP level differentiating ability when equal weight is given to sensitivity and specificity. 

Following J index value, the subjects were grouped in class 1 and class 2; those variables were compared with the Mann–Whitney test. 

A *p* value < 0.05 (two-tailed) was considered statistically significant. 

## 3. Results

We assayed semen levels of F_2_-IsoPs in a group of 192 men. Descriptive statistics for the variables considered (median and IQR) such as volume, sperm concentration, motility, normal morphology, viability and F_2_-IsoPs are shown in Table 1.

F_2_-IsoPs correlated negatively with sperm total progressive motility (r = −0.309, *p* < 0.01), rapid motility (r = −0.535, *p* < 0.01), normal morphology (r = −0.283, *p* < 0.01) and viability (r = −0.245, *p* < 0.01) and positively with slow motility (r = 0.218, *p* < 0.01). Semen parameters correlated positively with each other (data not shown).

Men were then grouped according to their reproductive status: fertile men (n = 45, 23% of total subjects), patients with idiopathic infertility (n = 41, 21.4% of total subjects), patients with urogenital infection (n = 52, 27.1% of total subjects) and patients with varicocele (n = 54, 28.1% of total subjects). 

The semen variables considered were compared in the four groups (Table 2).

**Figure 1:**
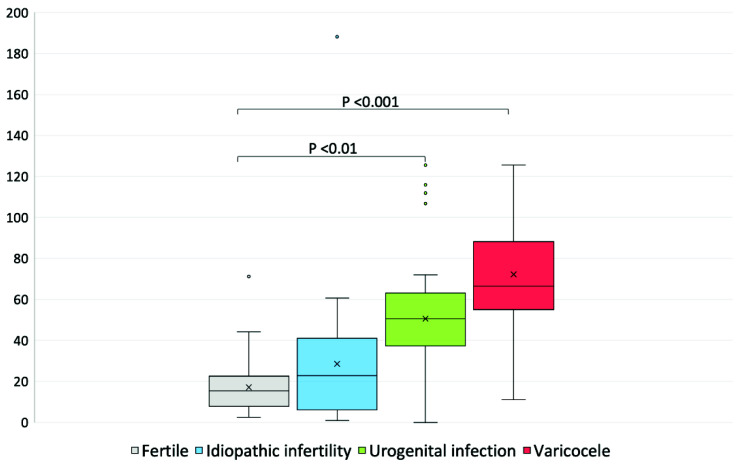
Box plot of the distribution of F_2_-IsoP concentrations (ng/mL) in fertile men and patients affected by idiopathic infertility, urogenital infections and varicocele.

The F_2_-IsoP concentration was significantly higher in the urogenital infection and varicocele groups than that observed in fertile individuals (Table 2, Figure 1).

**Figure 2:**
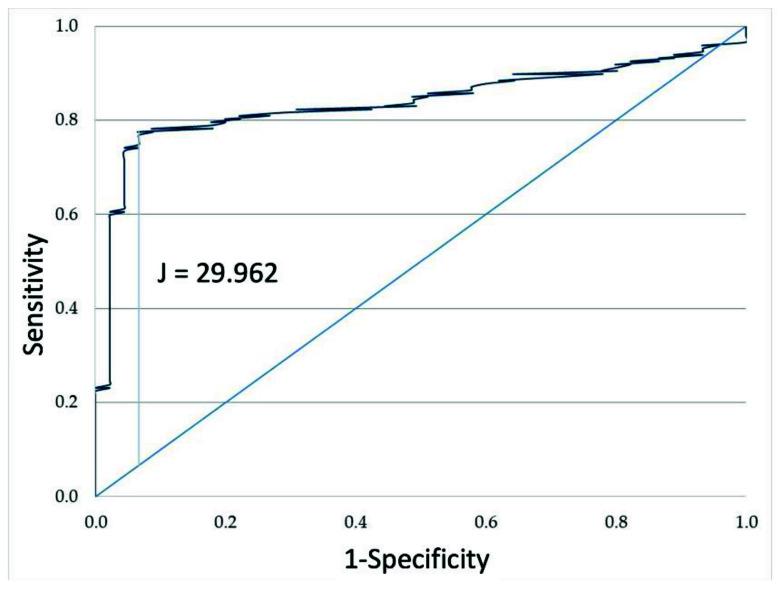
ROC curve of semen F_2_-IsoP levels in fertile (n. 45) and infertile (n. 147) subjects considered in this study.

A ROC curve and the best cut-off value according to the J index for F_2_-IsoP levels were calculated. The overall performance of the ROC test was quantified by computing the area under the curve, which for F_2_-IsoP levels was 0.839 (0.781–0.897 confidence interval, sensitivity: 0.776 and 1-specificity: 0.067; Figure 2). J index showed a value of 29.96 ng/mL.

Two different subgroups of subjects were identified on the basis of J value related to the F_2_-IsoP levels: class 1 identified by F_2_-IsoP levels < 29.96 ng/mL (n = 78 individuals, 39.1% of total subjects), and class 2 associated with F_2_-IsoP levels > 29.96 ng/mL (n = 114 individuals, 60.9% of total subjects). 

The semen variables in the two groups with different levels of F_2_-IsoPs indicated a good sperm quality (Table 3) in class 1 (F_2_-IsoP amount, 11.20 (14.93) ng/mL). Class 2 (F_2_-IsoP amount, 59.80 (27.23) ng/mL) showed a significant reduction in sperm concentration, total progressive and rapid motility, normal morphology and viability (Table 3). Class 1 included 1 patient with varicocele, 9 with urogenital infection, 24 with idiopathic infertility and 44 fertile men. 

Class 2 encompassed 53 patients with varicocele, 43 with urogenital infection, 17 with idiopathic infertility and 1 fertile man. 

## 4. Discussion

The aim of this paper was to assay semen levels of F_2_-IsoPs in fertile and infertile men to identify a cut-off value characterizing physiological and pathological concentrations of these compounds. 

It is well known that oxygenated arachidonic metabolites (i.e., F_2_-IsoPs) are regarded as in vivo markers of LPO [7].

Interestingly, oxidative stress and inflammation have been suggested to be involved in different pathologies related to male fertility, including varicocele, leukocytospermia, sexually transmitted diseases and bacterial prostatitis [18].

In previous studies, our group detected the presence of increased levels of isoprostanoids in the semen of infertile patients, particularly in subjects with varicocele [19]. F_2_-IsoP levels were dosed in a quite large population, and they correlated negatively with rapid and total sperm motility, normal morphology and viability, suggesting that these molecules might represent a marker of semen quality.

In this research, the defined J index for F_2_-IsoP level was 29.96 ng/mL, which could be considered the cut-off point under which the F_2_-IsoP levels represent a situation of normality in human semen while an increased concentration could be evaluated as an index of altered sperm quality in infertile patients.

Based on the results of the ROC curve and J index, we divided the studied cases into two classes according to their F_2_-IsoP amount (class 1, <29.96 ng/mL; class 2, >29.96 ng/mL). We observed that class 1 included all fertile men (except one) and 58.54% of patients with idiopathic infertility; in class 1, the semen parameters did not show significant reductions, highlighting a median of total sperm progressive motility equal to 40.00 (25.50) and rapid motility equal to 32.00 (19.00). Only infertile patients belonged to class 2, except one; a significant worsening of sperm quality was detected, particularly in total progressive and rapid sperm motility.

These observations indicated that the cut-off value is able to discriminate normal and altered semen parameters in terms of sperm concentrations, total and progressive motility, normal sperm morphology and viability.

F_2_-IsoP levels above 29.96 ng/mL seem to be related to the presence of pathologies, as observed in the semen of patients with varicocele and genitourinary infections and almost 50% of those with idiopathic infertility.

Previously, the level of 8-iso-PGF_2α_ was investigated in urine [20] as a marker of oxidative stress; in addition, van ‘t Erve et al. [6] reported an increase in 8-iso-PGF_2α_ levels in several inflammatory conditions. Recently, growth in F_2_-IsoP blood concentration was related to the prognosis of cardiac disease [21] and systemic sclerosis [22]. In the present research, the increased levels of F_2_-IsoPs detected in urogenital infections and varicocele groups can be explained with the association of these pathologies to the presence of high reactive oxygen species (ROS) levels [23] and inflammation. Idiopathic infertility is a peculiar condition in which an inflammatory status may not necessarily be involved.

Semen parameters are not sufficient to fully characterize pathological conditions; for this reason, the definition of a cut-off value and the ranges of F_2_-IsoPs could help to understand the severity of the condition and suggest appropriate interventions.

For this purpose, Krzyściak et al. [24] highlighted the importance of using the combination of oxidative–antioxidant markers as potential predictors of male infertility.

Moreover, these results would suggest a dual function of these molecules: high levels of F_2_-IsoPs represent an index of inflammation/oxidative stress, while levels < 29.96 ng/mL could be considered physiological and normally present in semen samples. Accordingly, the dual, harmful and beneficial, effect of oxidative stress on human health is well known [25].

Recently, a mild increase in semen F_2_-IsoP levels, lower than the cut-off point found in the present research, has been found to have a positive relationship with sperm DNA integrity and good embryo quality [15]. These observations support that the F_2_-IsoP concentration < 29.96 ng/mL is normal and can represent a marker of metabolic activity in human semen, as was also detected for F_4_-neuroprostanes, molecules derived from the oxidative metabolism of docosahexaenoic acid, which at defined levels are able to stimulate sperm capacitation [17].

## 5. Conclusions

In conclusion, the ROC curve related to seminal F_2_-IsoP level provided a specific and sensitive accuracy with a J index value of 29.96 ng/mL. In addition, by grouping patients based on pathology and control, ranges of F_2_-IsoP levels were identified.

Obviously, further large-scale studies including more parameters such as DNA fragmentation index, antioxidant system and other markers of OS should be performed in order to suggest seminal levels of F_2_-IsoP as a biomarker in the interpretation of the result of an individual test for infertility.

## Figures and Tables

**Table 1:** Median and IQR of semen variables in 192 individuals.

Vol (mL)	Sperm (10^6^/mL)	Progressive Motility (%)	Rapid Motility (%)	Slow Motility (%)	Normal Morphology (%)	Viability (%)	F_2_-IsoPs (ng/mL)
4.00 (1.50)	37.88 (68.98)	29.00 (21.00)	18.00 (20.00)	10.00 (11.00)	9.00 (9.00)	70.00 (22.25)	40.90 (44.64)

**Table 2:** Median (IQR) of semen variables in each categorized group (fertile, idiopathic infertility, urogenital infection and varicocele).

Variable	Fertile Men (F, n = 45)	Idiopathic Infertility (I, n = 41)	Urogenital Infections (U, n = 52)	Varicocele (V, n = 54)	Statistics
**Vol (mL)**	4.00 (1.30)	4.00 (1.50)	3.65 (1.15)	4.00 (0.98)	
**Sperm (10^6^/mL)**	110.00 (98.00)	12.00 (31.90)	24.50 (47.48)	38.50 (31.56)	F vs. I, U, V. *p* < 0.001 I vs. U. *p* < 0.05
**Progressive motility (%)**	51.00 (18.00)	28.00 (15.00)	23.00 (12.25)	24.50 (16.75)	F vs. I, U, V. *p* < 0.001
**Rapid motility (%)**	40.00 (10.00)	20.00 (13.00)	12.00 (12.25)	12.00 (6.75)	F vs. I, U, V. *p* < 0.001
**Slow motility (%)**	12.00 (11.00)	8.00 (9.00)	8.00 (8.50)	12.00 (10.75)	F vs. I. *p* < 0.05
**Normal morphology (%)**	15.00 (7.00)	7.00 (5.00)	6.00 (5.00)	8.00 (5.00)	F vs. I, U, V. *p* < 0.001
**Viability (%)**	85.00 (11.00)	65.00 (20.00)	60.00 (24.00)	65.00 (16.50)	F vs. I, U, V. *p* < 0.001
**F_2_-IsoPs** **(ng/mL)**	15.36 (14.41)	22.80 (31.44)	52.10 (24.62)	66.40 (31.91)	F vs. U. *p* < 0.01 F vs. V. *p* < 0.001

**Table 3:** Median (IQR) of semen variables of the considered subjects grouped following J value calculated for F_2_-IsoP level.

Variable	Class 1 (<29.96 ng/mL) (n = 78)	Class 2 (>29.96 ng/mL) (n = 114)
**Vol (mL)**	4.00 (1.50)	4.00 (1.40)
**Sperm (10^6^/mL)**	56.00 (98.43)	25.00 (40.00) **
**Progressive motility (%)**	40.00 (25.50)	23.00 (17.00) ***
**Rapid motility (%)**	32.00 (19.00)	12.00 (10.00) ***
**Slow motility (%)**	10.00 (13.00)	10.00 (10.00)
**Normal morphology (%)**	12.00 (7.00)	7.00 (6.00) *
**Viability (%)**	78.00 (20.00)	64.00 (20.00) *
**F_2_-IsoPs (ng/mL)**	11.20 (14.93)	59.80 (27.23) ***

* *p* < 0.05, ** *p* < 001, *** *p* < 0.001.

## Data Availability

The data presented in this study are available on request from the corresponding author. The data are not publicly available due to part of them are being used in other studies that have not yet been publicly published.
